# Editorial: Bridging quantitative imaging and artificial intelligence methods in preclinical and clinical oncology

**DOI:** 10.3389/fonc.2023.1272030

**Published:** 2023-09-01

**Authors:** Jacobus FA. Jansen, Ramesh Paudyal, Yousef Mazaheri, Amita Shukla-Dave

**Affiliations:** ^1^ Department of Radiology and Nuclear Medicine, Maastricht University Medical Center+, Maastricht, Netherlands; ^2^ School for Mental Health and Neuroscience (MHeNs), Maastricht University, Maastricht, Netherlands; ^3^ Department of Electrical Engineering, Eindhoven University of Technology, Eindhoven, Netherlands; ^4^ Department of Medical Physics, Memorial Sloan Kettering Cancer Center, New York, NY, United States; ^5^ Department of Radiology, Memorial Sloan Kettering Cancer Center, New York, NY, United States

**Keywords:** cancer, artificial intelligence, radiomics, quantitative image analyses, personalized medicine

Cancer continues to be a significant global health challenge, demanding innovative approaches for early diagnosis and personalized treatment. Quantitative imaging is an emerging field in oncology that enables accurate quantification of physiological parameters from a target area (1). Recent advancements in quantitative imaging aided with artificial intelligence (AI) have gained interest in oncological imaging and can play an important role in improving personalized oncological care (2), for example, for diagnosis (3) or prediction of treatment outcome (4). AI can be implemented in several stages of the imaging workflow, including data acquisition, processing, and analysis (5). Additionally, the radiomics approach is very popular, which enables the extraction of large numbers of features from images using data-characterization algorithms (6). The five manuscripts included in this Research Topic cover various aspects of the application of advanced technologies such as radiomics, deep learning, and machine learning using imaging data from multiple modalities, including computed tomography (CT), positron emission tomography (PET), magnetic resonance imaging (MRI), and ultrasound (US) to improve cancer diagnosis, prediction of specific gene mutations, cancer subtypes, and early recurrence as well as the development of an effective data preparation pipeline. In this editorial we will briefly discuss these five studies that exemplify the successful application of these techniques in different cancer types, each demonstrating the value of these cutting-edge technologies in the field of oncology.

The study by Huo et al. delved into the realm of lung adenocarcinoma (LADC) and aimed to identify epidermal growth factor receptor (EGFR)-mutation subtypes using CT-based radiomics signatures combined with clinical and CT morphological features. They successfully developed three combined models with high area under the curve (AUC) values in the validation cohorts by analyzing a large dataset of 608 LADC patients. These models proved to be valuable auxiliary tools for predicting EGFR-mutation subtypes, contributing to personalized treatment decisions for LADC patients.

Zhao et al. expanded the application of machine learning models to distinguish between LADC and lung squamous cell carcinoma (LUSC). By incorporating clinical factors, laboratory metrics, and PET/CT radiomic features, they developed Random Forest (RF) and Support Vector Machine (SVM) models that exhibited excellent performance in identifying the two cancer types. These noninvasive predictive tools are particularly useful when biopsies are not feasible or have failed, empowering clinicians to make informed treatment decisions based on accurate classification.

The objective of the study by Zheng et al. was to enhance the diagnosis of follicular thyroid carcinoma (FTC) through the development of a computer-aided diagnosis (CAD) system based on deep learning and thyroid ultrasonography. The CAD system outperformed junior and senior radiologists in accuracy, recall, precision, and F1-score, demonstrating its potential as a reliable reference for preoperative FTC diagnosis. This innovative approach could pave the way for a faster and more accessible screening method for FTC, ultimately improving patient outcomes.

Moving beyond lung and thyroid cancer, Zhang et al. explored the potential of a multi-parametric MRI-based radiomics nomogram in predicting early recurrence (ER) of small hepatocellular carcinoma (HCC) after radiofrequency ablation (RFA). They successfully developed a radiomics model based on preoperative T1- and T2- weighted and contrast-enhanced MRI by analyzing data from 90 patients with small HCC. The model showed high predictive performance and, when combined with clinical risk factors, demonstrated clinical usefulness in identifying early recurrence, allowing for timely intervention and improved patient management.

Finally, Krajnc et al. introduced the concept of machine learning-driven data preparation (MLDP) to optimize data preparation before building cancer prediction models. The proposed MLDP was validated on glioma and prostate single-center cohorts and a dual-center diffuse large B-cell lymphoma cohort. Through evolutionary algorithms and hyperparameter optimization, MLDP tailored data preparation pipelines for each dataset, significantly improving the performance of predictive models, especially for random forest and support vector machine models. The effectiveness of ML-driven data preparation was demonstrated in both single and multi-centric cancer settings, highlighting the potential for widespread application and enhanced model accuracy.

In conclusion, the five studies discussed in this editorial collectively demonstrate the power of radiomics and AI techniques in cancer research and clinical practice. From the identification of cancer subtypes and distinguishing between different types of cancer to predicting early recurrence and optimizing data preparation, these advanced computational approaches have proven to be invaluable tools in the fight against cancer. As the field continues to progress, it is essential to foster collaboration between researchers, clinicians, and data scientists to harness the full potential of radiomics and AI to benefit cancer patients worldwide. Furthermore, the concept of quantitative multimodality imaging which enables simultaneous quantification of physiological parameters from multiple images (7), holds enormous potential to provide clinicians with valuable information for making informed decisions and ultimately improving patient outcomes. By leveraging these technologies, we can usher in a new era of precision oncology, where early diagnosis and personalized treatment strategies become the standard, ultimately leading to improved patient outcomes and a brighter future in the battle against cancer.

## Author contributions

JJ: Writing – original draft, Writing – review & editing. RP: Writing – review & editing. YM: Writing – review & editing. AS-D: Writing – review & editing.
